# Early atherosclerosis and cardiac autonomic responses to mental stress: a population-based study of the moderating influence of impaired endothelial function

**DOI:** 10.1186/1471-2261-10-16

**Published:** 2010-03-29

**Authors:** Nadja Chumaeva, Mirka Hintsanen, Taina Hintsa, Niklas Ravaja, Markus Juonala, Olli T Raitakari, Liisa Keltikangas-Järvinen

**Affiliations:** 1 Institute of Behavioural Sciences, University of Helsinki, P.O. Box 9, FIN-00014 Helsinki, Finland; 2 Division of Medical Problems of Cell Biology, Institute of Cell Biophysics, Institutional Street 3, Pushchino, Moscow region, 142290 Russia; 3 Center for Knowledge and Innovation Research, Helsinki School of Economics, P.O. Box 1210, 00101 Helsinki, Finland; 4 Research Centre of Applied and Preventive Cardiovascular Medicine, University of Turku, Kiinanmyllynkatu 10, 20520 Turku, Finland; 5 Department of Clinical Physiology, University of Turku and Turku University Hospital, P.O. Box 52, 20521 Turku, Finland

## Abstract

**Background:**

Acute mental stress may contribute to the cardiovascular disease progression via autonomic nervous system controlled negative effects on the endothelium. The joint effects of stress-induced sympathetic or parasympathetic activity and endothelial function on atherosclerosis development have not been investigated. The present study aims to examine the interactive effect of acute mental stress-induced cardiac reactivity/recovery and endothelial function on the prevalence of carotid atherosclerosis.

**Methods:**

Participants were 81 healthy young adults aged 24-39 years. Preclinical atherosclerosis was assessed by carotid intima-media thickness (IMT) and endothelial function was measured as flow-mediated dilatation (FMD) using ultrasound techniques. We also measured heart rate, respiratory sinus arrhythmia (RSA), and pre-ejection period (PEP) in response to the mental arithmetic and speech tasks.

**Results:**

We found a significant interaction of FMD and cardiac RSA recovery for IMT (p = 0.037), and a significant interaction of FMD and PEP recovery for IMT (p = 0.006). Among participants with low FMD, slower PEP recovery was related to higher IMT. Among individuals with high FMD, slow RSA recovery predicted higher IMT. No significant interactions of FMD and cardiac reactivity for IMT were found.

**Conclusions:**

Cardiac recovery plays a role in atherosclerosis development in persons with high and low FMD. The role of sympathetically mediated cardiac activity seems to be more important in those with impaired FMD, and parasympathetically mediated in those with relatively high FMD. The development of endothelial dysfunction may be one possible mechanism linking slow cardiac recovery and atherosclerosis via autonomic nervous system mediated effect.

## Background

Mental stress has been shown to be a risk factor for atherosclerosis [1]. Acute mental stress may induce myocardial infarction [2] or sudden cardiac death [3]. It has been found to impair the parameters of endothelial health, reducing flow-mediated dilatation (FMD) [4,5]. Brachial FMD is an adequate non-invasive measure of endothelial function [6] and reduced brachial FMD reflects endothelial dysfunction [6,7]. Endothelial dysfunction is a marker of cardiovascular risk [6,7] and may be considered as an indicator of atherosclerotic events in later stages in life [8]. Brachial FMD as well as carotid intima-media thickness (IMT) are important non-invasive markers of subclinical atherosclerosis [7,9]. Increased carotid IMT correlates with coronary atherosclerosis [10] and increased IMTs have been found among individuals with impaired brachial FMD [11].

Mechanisms through which mental stress induces harmful changes in vascular system functioning and influences atherosclerosis development are not fully clear. A novel hypothesis has considered atherosclerosis as a neurogenic phenomenon manifested by the autonomic nervous system (ANS) dysfunction [12]. It has been suggested that chronic stress may promote atherogenesis through the mechanism of autonomic neuropathy-caused sympathetic hyperactivity [12].

Sympathetic hyperactivity impairs the ANS control of the cardiovascular system [13]. It has been considered that suppressed ANS regulation may be related to atherosclerotic processes through the negative ANS-mediated effect on the endothelium [14]. Using a nonhuman primate model of atherogenesis, it has been found that psychosocial stress can alter the autonomic balance towards a state of sympathetic arousal leading to the development of coronary heart disease (CHD), perhaps through impairing endothelial function [15] and intensification of endothelium-mediated atherogenic processes [16].

The reactivity hypothesis suggests that elevated cardiovascular reactivity evoked by the psychological stress factors is a risk for the development of hypertension, atherosclerosis, and CHD [17]. In addition, the disability of the cardiovascular system to recover from psychological stress has been mentioned in several studies as a risk factor for the development of cardiovascular disorders [18,19].

It has been shown that acute mental stress induces changes in heart rate (HR) [20]. Many studies of cardiac reactivity have used pre-ejection period (PEP) as an adequate noninvasive indicator of cardiac sympathetic regulation [21,22] and respiratory sinus arrhythmia (RSA) as an index of parasympathetic control of HR [23].

Psychological risk factors are highly associated with the measures of cardiovascular ANS reactivity [20] and, on the other hand, may accelerate endothelium-mediated atherogenesis [15,16]. In addition, associations between acute mental stress and IMT have recently been shown [24], and increased carotid IMT levels have been found among healthy young adults who have impaired brachial FMD [11]. It can be suggested that acute mental stress may contribute to the cardiovascular disease progression via ANS-controlled negative effects on the endothelium, predisposing some individuals to an autonomic imbalance that may be harmful to endothelial function and, therefore, may represent a negative prognostic factor for atherosclerosis. There is little knowledge on this hypothesis so far.

The joint effects of acute mental stress-induced sympathetic or parasympathetic activity and endothelial function on atherosclerosis development have not been studied. The aim of this study was to examine the interactive effect of acute stress-induced cardiac reactivity/recovery and endothelial function, measured in terms of FMD, on the prevalence of preclinical atherosclerosis assessed by IMT in young healthy adults.

## Methods

### Study population

The participants were 100 healthy men and women aged 24-39 years (in 2001), who were participating in the prospective multicenter epidemiological Cardiovascular Risk in Young Finns (CRYF) study [25]. Since 1980, the CRYF study has been monitoring the development of risk factors for coronary heart disease at intervals of 3 or 5 years. The CRYF study originally included a total of 3596 healthy Finnish children, adolescents, and young adults at baseline in 1980. The design of the study and the sample have recently been described [25]. Of the original CRYF sample, 2109 participants were examined in 2001 and cardiovascular risk factor and ultrasound measurements were performed on them. The stress testing (public speaking task and mental arithmetic task) was administered for 95 healthy young adults two years before the ultrasound measurements (in 1999).

The present sample (originally a total of 100 subjects) was randomly selected from the subjects who participated in the psychological study in 1997. The participants were invited to the stress testing from the urban and rural districts of Finland within a 100 km radius from Helsinki (n = 382). Ninety-five subjects entered in the psychophysiological testing in 1999. In the present study, complete data on psychophysiological reactivity/recovery testing and ultrasound measurements were obtained from 81 participants. They comprised the final sample of this study.

The study followed the guiding principles of the Helsinki Declaration and was approved by the Ethical Committee of the University of Helsinki and by the Joint Ethical Committee of the University of Turku and Turku University Hospital. All subjects gave their written, informed consent in 1999 and in 2001.

### Cardiac measures

We measured heart rate (HR), respiratory sinus arrhythmia (RSA; indicator of the parasympathetic control of HR), and pre-ejection period (PEP; indicator of the sympathetic control of HR).

Electrocardiogram (ECG) and the first derivative of the pulsatile impedance signal (dZ/dt) were measured continuously during the experiment with Minnesota Impedance Cardiograph Model 304B (Surcom Inc., Minneapolis, MN), using the standard tetrapolar band electrode configuration [26]. The ECG and dZ/dt signals were sampled continuously at 500 Hz via a 12-bit 8-channel A/D converter, and stored on the hard disk of a PC for later analysis. On-line data reduction was performed with custom-programmed Labview data acquisition software (National Instruments Co., Austin, TX).

### Ultrasound imaging

Ultrasound studies of the carotid and brachial arteries were performed using Sequoia 512 ultrasound mainframes (Acuson, Mountain View, CA, USA) with 13.0 MHz linear array transducer, as previously described [11,27]. To assess intra-individual reproducibility of ultrasound measurements, 57 subjects were re-examined 3 months after the initial visit (2.5% random sample) [11].

### Carotid intima-media thickness, IMT

Carotid IMT was measured on the posterior (far) wall of the left carotid artery. At least four measurements were taken approximately 10 mm proximal to the bifurcation to derive mean carotid IMT. The between-visit coefficient of variation of IMT measurements was 6.4% [11].

### Brachial flow-mediated dilatation, FMD

To assess brachial FMD, the left brachial artery diameter was measured both at rest and during reactive hyperemia. Increased flow was induced by inflation of a pneumatic tourniquet placed around the forearm to a pressure of 250 mmHg for 4.5 min, followed by a release [11]. Arterial diameter was measured at end-diastole at a fixed distance from an anatomic marker at rest and 40, 60 and 80 s after cuff release. The vessel diameter in scans after reactive hyperemia was expressed as the percentage relative to resting scan. The average of three measurements at each time point was used to derive the maximum FMD (the greatest value between 40 to 80 s). The between-visit coefficient of variation for brachial diameter was 3.2% and for FMD 26.0% [11].

### Experimental procedure

Before the experiment, the subjects were informed about the general nature of the study, and gave their written informed consent. Each participant was studied using a standardized computer-controlled experimental session. The experiment lasted for about 180 min, and the experimental procedure started at the same time of the day (9:00 am) for all participants. The subjects were instructed to abstain from caffeine and cigarettes consumption for 12 hr before the experimental procedure. The experiment was conducted in a sound-attenuated room equipped with a computer for stimulus presentation, and with a video system for monitoring and communication. The experiment consisted of five tasks: (1) emotion-evoking picture viewing, (2) acoustic startle stimuli, (3) a mental arithmetic task, (4) a reaction-time task, and (5) a public-speaking task with three different scenarios. The participants were alone in the room during the tasks, with the exception of the public speaking task. Two experimenters were present as an audience during the public speaking task. The experiment started and ended with a resting period of 10 min. Each of the experimental tasks was followed by an 8 min resting period. The mental arithmetic task and the speech task were used in the current study. The tasks have been described previously in more detail [28]. The other tasks were omitted because they evoked very different reactivity profiles compared to the two selected tasks. In the present study, both the mental arithmetic and public speaking tasks evoked an increase in HR and a decrease in RSA and PEP, which is in line with many previous studies [20,29]. The public speaking and mental arithmetic tasks have been shown to evoke stable and relatively high cardiac autonomic responses [29,30].

### Data treatment

The interbeat intervals (IBIs), in ms, were determined from the ECG signal and deviant IBI values were identified using a 20% change from the previous IBI as a criterion. They were corrected according to the guidelines of [31]. The beat-to-beat IBI data were transformed to equidistant IBI time series with 200 ms intervals using the weighted-average interpolation method of [32]. The spectral analyses were conducted on 60-second segments of the heart period series. The mean and trend were removed from each IBI segment to exclude long-term changes in the time series. The impedance data were ensemble averaged within 60-s blocks. The Q-waves and the B-points were determined by a careful visual examination with the aid of a self-programmed computer program similar to that described by [33].

RSA was computed separately for each 60-s data segment. The logarithm of the variance (ms^2^) within the frequency band associated with respiration (i.e., 0.12-0.40 Hz) was summed to index RSA. PEP, in ms, was calculated as the interval between the Q wave of the ECG and the B-point of the dZ/dt waveform. HR, in bpm, was computed for 60-s intervals from the mean IBI.

Mean HR, RSA, and PEP values were calculated for each participant across each minute during the tasks. For the resting periods, data were averaged across minutes 6, 7, and 8 during the 10-min initial baseline and last rest baseline. Task HR, RSA, and PEP data were averaged across the 6 min of the mental arithmetic task and across the 3 min of the speech task. The mean of three speeches was calculated and speech delivery period was used in the present study. Reactivity scores were calculated by subtracting the initial mean baseline value from average task value (for each task separately). Averaged value for the reactivity score across the two tasks was calculated finally. Recovery scores were computed by subtracting the initial mean baseline value from the average value during the resting periods after the mental arithmetic and speech tasks.

### Statistical analysis

The interaction between cardiac autonomic measures and FMD in predicting carotid IMT was tested by linear regression analyses using SPSS Version 16.0. All regression models involving interactions between FMD and cardiac reactivity/recovery were conducted separately for reactivity and recovery terms, and separately for HR, RSA, and PEP. Main effects of age, sex, brachial baseline diameter, FMD, and the reactivity/recovery measure in question were included in the regression models.

## Results

Table 1 shows the characteristics of the study variables.

**Table 1:** Characteristics of study participants

Variable	Min	Max	Mean	(SD)	N
Age in 2001, years	24.00	39.00	30.89	4.92	81
Baseline brachial diameter, mm	2.36	5.32	3.53	0.62	74
Flow-mediated dilatation, FMD, %	-0.26	23.43	6.78	5.00	74
Carotid intima-media thickness, IMT, mm	0.43	0.96	0.59	0.09	81
Baseline HR, bpm	46.30	94.48	71.63	9.30	75
Baseline RSA, log ms^2^	2.17	3.30	2.84	0.23	76
Baseline PEP, ms	74.00	128.67	96.94	11.17	75
HR reactivity score, bpm	-4.23	46.28	16.33	10.74	72
RSA reactivity score, log ms^2^	-0.88	0.34	-0.11	0.24	73
PEP reactivity score, ms	-44.33	5.30	-14.80	10.65	70
HR recovery score, bpm	-14.02	12.37	-2.03	4.84	71
RSA recovery score, log ms^2^	-0.49	0.64	0.01	0.18	72
PEP recovery score, ms	-17.33	17.00	2.20	6.27	70

HR = heart rate (bpm); RSA = respiratory sinus arrhythmia (log ms^2^); PEP = pre-ejection period (ms).

### Associations between the study parameters

Linear regression analyses showed that baseline HR, baseline RSA, and baseline PEP were unrelated to IMT (N = 75, *p *= 0.893; N = 76, *p *= 0.363; N = 75, *p *= 0.324, respectively) and FMD (N = 69, *p *= 0.114; N = 70, *p *= 0.969; N = 69, *p *= 0.177, respectively). In addition, FMD was not related to IMT (N = 74, *p *= 0.207). HR, RSA, and PEP reactivity scores were not associated with FMD (N = 66, *p *= 0.805; N = 67, *p *= 0.615; N = 65, *p *= 0.152, respectively). Likewise, HR, RSA, and PEP recovery scores were unrelated to FMD levels (N = 65, *p *= 0.982; N = 66, *p *= 0.709; N = 65, *p *= 0.175, respectively).

### Interactions between the cardiac measures and FMD in predicting IMT

Table 2 shows the results of linear regression analyses examining the interactions of cardiac reactivity and recovery with FMD for IMT. No significant interaction effect of FMD and cardiac reactivity on IMT was found (Table 2).

**Table 2:** Linear regression examining the interactive effects of physiological reactivity/recovery and flow-mediated dilatation on IMT (N = 61)

	*R*^2^	*Adjusted* *R*^2^**	*R*^2 ^change***	*p-value*
**REACTIVITY**				
HR reactivity × FMD*	0.38	0.31	0.002	0.704
RSA reactivity × FMD*	0.33	0.25	0.020	0.214
PEP reactivity × FMD*	0.46	0.40	0.010	0.321

**RECOVERY**				
HR recovery × FMD*	0.30	0.23	0.000	0.886
RSA recovery × FMD*	0.38	0.26	0.056	***0.037***
PEP recovery × FMD*	0.43	0.36	0.087	***0.006***

*Each interaction is examined in a separate analysis including the main effects of sex, age, brachial baseline diameter, FMD, and the reactivity/recovery measure in question, but the main effects are not presented in the Table.

**Calculated for the whole model.

***Refers to the interaction term.

HR = heart rate (bpm); RSA = respiratory sinus arrhythmia (log ms^2^); PEP = pre-ejection period (ms); FMD = flow-mediated dilatation (%).

A significant RSA recovery × FMD interaction (*p *= 0.037, N = 61) in predicting IMT was found. Further linear regression analyses conducted separately for high (N = 31) and low (N = 30) FMD individuals (median split) showed that, among participants with higher FMD, better RSA recovery predicted lower IMT (β = 0.411, *p *= 0.022, N = 31). No associations were found in low FMD participants (β = -0.265, *p *= 0.157, N = 30).

The PEP recovery × FMD interaction in predicting IMT was also significant (*p *= 0.006; N = 61) (Table 2). Linear regression analyses performed separately in high (N= 31) and low (N = 30) FMD groups (median split) showed that, among low FMD individuals, better PEP recovery predicted lower IMT (β = 0.517, *p *= 0.003, N = 30). This association was non-significant in high FMD subjects (β = 0.075, *p *= 0.690, N = 31). The HR recovery × FMD interaction for IMT was non-significant (*p *= 0.886; N = 61) (Table 2).

## Discussion

The results of the current study showed no significant interactive effect of FMD and cardiac reactivity on IMT. Previously, recovery responses have been reported to be better predictors of cardiovascular risk than stress-induced reactivity responses [18,19,34]. In line with this, significant RSA recovery × FMD interactions in predicting IMT, and a PEP recovery × FMD interaction for IMT, were found in our study. Better RSA recovery responses (mediated by parasympathetic activity) after acute mental stress were shown to be related to lower IMT among participants with higher FMD. These results are in agreement with our previous findings demonstrating that better cardiac recovery after acute mental stress is associated with less preclinical atherosclerosis among healthy men and women [24]. Other studies have also found that impaired parasympathetic control is related to an increased risk for early atherosclerosis development in healthy individuals [35]. In addition, slower PEP recovery (mediated by sympathetic activity) after acute mental stress was shown in our study to be related to higher IMT among subjects with lower FMD. These results are in line with the findings that acute mental stress may induce vascular changes both among healthy individuals and among patients with vascular disease [36]. In addition, several studies have reported negative relations between vascular reactions in response to acute mental stress and endothelial function [37,38].

It has been hypothesized that chronic stress can speed up atherogenesis through the mechanism of autonomic imbalance [12], and acute mental stress is associated with sympathetic activation [39]. In the current study, the relationship of slower PEP recovery to acute stress in combination with reduced FMD with higher IMT suggests that slow sympathetic recovery increases the risk of atherosclerosis development among individuals with impaired endothelial function, but not among subjects with normal endothelial function. It is in agreement with the response-to-injury model of atherosclerosis suggesting that impaired endothelial function is a first step of atherosclerosis development [40]. Our results are also in line with the findings that abnormalities in ANS regulation of the cardiovascular system [41,42], as well as enhanced endothelium-mediated atherogenic processes [43,44], are typical for patients with cardiovascular and metabolic diseases.

However, the mechanisms underlying the relationships between the ANS and endothelial systems and their joint influence on atherosclerosis progression are not fully clear. One of the recent hypotheses has considered atherosclerosis as a vascular symptom of ANS dysregulation induced by age, smoking, hypertension, dyslipidemia, and diabetes [12]. This hypothesis explains the atherogenic processes as a result of a global ANS imbalance towards to a state of enhanced sympathetic activity [12]. In addition, local atherosclerotic tissue alterations, such as endothelial dysfunction, have been suggested to result from neurogenic adventitial stress mediated by ANS dysregulation [12]. The delayed cardiovascular recovery may be considered as a result of a continual influence of acute mental stressors on the cardiovascular system [45].

Our results showed that RSA recovery following acute mental stress was related to atherosclerosis development in young adults with a high level of FMD, whereas, for PEP recovery the association with atherosclerosis development emerged in participants with low FMD. The reason for this discrepancy is not readily apparent. Our results showing delayed RSA and PEP recovery may reflect the autonomic imbalance. We can suggest that ANS dysregulation is a very essential independent reason for cardiovascular health problems, and it may increase a risk of cardiovascular diseases independently on endothelium functioning. In line with this suggestion, ANS imbalance has been shown to be associated with ineffective cardiovascular functioning or with a risk of atherosclerosis even if the endothelium works properly, i.e., among healthy individuals [35]. On the other hand, we can suggest, that harmful influence of ANS dysregulation may be accelerated in impaired endothelium (reflected by low FMD in the present study). Our results reflect both these situations. In our study, slow RSA recovery was shown to be related to an increased IMT in normal endothelium, whereas slow PEP recovery was related to higher IMTs in impaired endothelium. These results are in line with the findings, that elevated sympathetic activity [41,46] and impaired parasympathetic control [35] have been shown to exert a harmful effect on cardiovascular functioning [41] or to be related with atherosclerotic processes [35] in both healthy individuals and in individuals with cardiovascular disease states. Cardiac recovery seems to play an important role in atherosclerosis development in persons with high and low FMD; however, we can suggest different impact of sympathetic and parasympathetic nervous system in atherosclerosis risk in individuals with normal or impaired endothelium. Thus, the role of sympathetically mediated cardiac activity seems to be more important in those with impaired FMD, and parasympathetically mediated in those with relatively high FMD.

One possible mechanism linking ANS-mediated poor cardiac recovery and atherosclerosis may be the development of endothelial dysfunction. Accordingly, the association of endothelial dysfunction and slow HR recovery has recently been shown [47]; and endothelial dysfunction has been found to be associated with increased carotid IMT [11]. However, in a recent study, the contribution of the sympathetic nervous system to the impairment of endothelial function after acute mental stress has been questioned, and an endothelin-A receptor mechanism has been suggested to play a role in the endothelial dysfunction-mediated relationship of mental stress with atherosclerosis development [5].

### Methodological considerations

There are some limitations in the present study. First, we did not measure blood pressure responses; thus, we cannot generalize our results to vascular reactivity and cannot make conclusions regarding the reactivity hypothesis that mainly concentrates on vascular reactivity.

Second, we did not measure respiration; thus, we cannot be sure that respiration fell within the frequency band used to compute RSA estimates. However, uncorrected RSA has recently been shown to be viable in indexing within-subject changes in the parasympathetic control of HR in the majority of stress studies [48]. It has been suggested that during the stable experimental conditions with constant respiratory parameters, the absence of respiratory assessment may not preclude group contrasts in well-specified populations with known patterns of respiration and large-amplitude RSA [49].

Third, the present analysis was conducted in participants aged 24 to 39 years. Our results cannot be generalized to older individuals with more manifest atherosclerosis. Finally, the ultrasound and cardiac autonomic measurements were conducted only once, thereby precluding conclusions concerning the progression of atherosclerosis.

In addition, the absence of blood pressure measurement is a relevant limitation of the study. Therefore, we cannot exclude the possibility of confounding.

## Conclusions

Our results suggest that alterations in ANS functioning, characterized by a decreased parasympathetic control in combination with normal endothelial function or increased sympathetic activity in combination with impaired endothelial function, are associated to increased risk of atherosclerosis progression in young healthy individuals. Cardiac recovery seems to play an important role in atherosclerosis development in individuals with high and low FMD but the role of sympathetically mediated cardiac activity seems to be more important in those with impaired FMD, and parasympathetically mediated in those with relatively high FMD.

The present study is the first to demonstrate that the development of endothelial dysfunction may be one possible mechanism linking slow cardiac recovery and atherosclerosis via ANS pathways. Our results suggest that psychophysiological risk factors may be associated with the development of atherosclerosis via interactions between the endothelial function and ANS regulation.

## Competing interests

The authors declare that they have no competing interests.

## Authors' contributions

LK-J and OTR were responsible for planning the study. NC was responsible for the data analysis and statistics. MH helped with data analysis and statistics. NC and NR have made substantial contributions to conception and design and LK-J, OTR, NR and MJ have made contribution to collecting and acquisition of data. OTR, LK-J and TH participated in the study design and coordination. All authors contributed in interpretation of data. All authors have been involved in drafting the manuscript and revising it. NC and MH had the main responsibility of the manuscript writing. All authors have read and given final approval of the version to be published.

## Pre-publication history

The pre-publication history for this paper can be accessed here:

http://www.biomedcentral.com/1471-2261/10/16/prepub

## References

[B1] Rozanski A Blumenthal JA Kaplan J Impact of psychological factors on the pathogenesis of cardiovascular disease and implications for therapy Circulation 1999 99 2192 2217 10217662 10.1161/01.cir.99.16.2192

[B2] Mittleman MA Maclure M Sherwood JB Mulry RP Tofler GH Jacobs SC Friedman R Benson H Muller JE Triggering of acute myocardial infarction onset by episodes of anger: determinants of Myocardial Infarction Onset Study Investigators Circulation 1995 92 1720 1725 7671353 10.1161/01.cir.92.7.1720

[B3] Leor J Poole WK Kloner RA Sudden cardiac death triggered by an earthquake N Engl J Med 1996 334 413 419 10.1056/NEJM199602153340701 8552142

[B4] Gottdiener JS Kop WJ Hausner E McCeney MK Herrington D Krantz DS Effects of mental stress on flow-mediated brachial arterial dilation and influence of behavioral factors and hypercholesterolemia in subjects without cardiovascular disease Am J Cardiol 2003 92 687 691 10.1016/S0002-9149(03)00823-3 12972107

[B5] Spieker LE Hürlimann D Ruschitzka F Corti R Enseleit F Shaw S Hayoz D Deanfield JE Lüscher TF Noll G Mental stress induces prolonged endothelial dysfunction via endothelin-A receptors Circulation 2002 105 2817 2820 10.1161/01.CIR.0000021598.15895.34 12070106

[B6] Celermajer DS Sorensen KE Gooch VM Spiegelhalter DJ Miller OI Sullivan ID Lloyd JK Deanfield JE Non-invasive detection of endothelial dysfunction in children and adults at risk of atherosclerosis Lancet 1992 340 1111 1115 10.1016/0140-6736(92)93147-F 1359209

[B7] Cohn JN Quyyumi AA Hollenberg NK Jamerson KA Surrogate markers for cardiovascular disease: functional markers Circulation 2004 109 Suppl 1 IV31 46 15226249 10.1161/01.CIR.0000133442.99186.39

[B8] Oliver JJ Webb DJ Noninvasive assessment of arterial stiffness and risk of atherosclerotic events Arterioscler Thromb Vasc Biol 2003 23 554 566 10.1161/01.ATV.0000060460.52916.D6 12615661

[B9] O'Leary DH Polak JF Intima-media thickness: a tool for atherosclerosis imaging and event prediction Am J Cardiol 2002 90 18L 21L 10.1016/S0002-9149(02)02957-0 12459422

[B10] Burke GL Evans GW Riley WA Sharrett AR Howard G Barnes RW Rosamond W Crow RS Rautaharju PM Heiss G Arterial wall thickness is associated with prevalent cardiovascular disease in middle-aged adults: the Atherosclerosis Risk in Communities (ARIC) Study Stroke 1995 26 386 391 7886711 10.1161/01.str.26.3.386

[B11] Juonala M Viikari J Laitinen T Marniemi J Helenius H Rönnemaa T Raitakari OT Interrelations between brachial endothelial function and carotid intima-media thickness in young adults: the Cardiovascular Risk in Young Finns study Circulation 2004 110 2918 2923 10.1161/01.CIR.0000147540.88559.00 15505080

[B12] Marwah RS Doux JD Lee PY Yun AJ Is atherosclerosis a neurogenic phenomenon? Med Hypotheses 2007 69 884 887 10.1016/j.mehy.2007.01.066 17400398

[B13] Dekker JM Crow RS Folsom AR Hannan PJ Liao D Swenne CA Schouten EG Low heart rate variability in a 2-minute rhythm strip predicts risk of coronary heart disease and mortality from several causes: the ARIC Study. Atherosclerosis Risk In Communities Circulation 2000 102 1239 1244 10982537 10.1161/01.cir.102.11.1239

[B14] Harris KF Matthews KA Interactions between autonomic nervous system activity and endothelial function: a model for the development of cardiovascular disease Psychosom Med 2004 66 153 164 10.1097/01.psy.0000116719.95524.e2 15039499

[B15] Strawn WB Bondjers G Kaplan JR Manuck SB Schwenke DC Hansson GK Shively CA Clarkson TB Endothelial dysfunction in response to psychosocial stress in monkeys Circ Res 1991 68 1270 1279 2018991 10.1161/01.res.68.5.1270

[B16] Manuck SB Kaplan JR Adams MR Clarkson TB Effects of stress and the sympathetic nervous system on coronary artery atherosclerosis in the cynomolgus macaque Am Heart J 1988 116 328 333 10.1016/0002-8703(88)90110-X 2899392

[B17] Manuck SB Cardiovascular reactivity in cardiovascular disease: "once more unto the breach" Int J Behav Med 1994 1 4 31 10.1207/s15327558ijbm0101_2 16250803

[B18] Linden W Earle TL Gerin W Christenfeld N Physiological stress reactivity and recovery: conceptual siblings separated at birth? J Psychosom Res 1997 42 117 135 10.1016/S0022-3999(96)00240-1 9076640

[B19] Schwartz AR Gerin W Davidson KW Pickering TG Brosschot JF Thayer JF Christenfeld N Linden W Toward a causal model of cardiovascular responses to stress and the development of cardiovascular disease Psychosom Med 2003 65 22 35 10.1097/01.PSY.0000046075.79922.61 12554813

[B20] Cacioppo JT Malarkey WB Kiecolt-Glaser JK Uchino BN Sgoutas-Emch SA Sheridan JF Berntson GG Glaser R Heterogeneity in neuroendocrine and immune responses to brief psychological stressors as a function of autonomic cardiac activation Psychosom Med 1995 57 154 164 7792374 10.1097/00006842-199503000-00008

[B21] Cacioppo JT Berntson GG Binkley PF Quigley KS Uchino BN Fieldstone A Autonomic cardiac control. II. Noninvasive indices and basal response as revealed by autonomic blockades Psychophysiology 1994 31 586 598 10.1111/j.1469-8986.1994.tb02351.x 7846219

[B22] Cacioppo JT Uchino BN Berntson GG Individual differences in the autonomic origins of heart rate reactivity: the psychometrics of respiratory sinus arrhythmia and pre-ejection period Psychophysiology 1994 31 412 419 10.1111/j.1469-8986.1994.tb02449.x 10690921

[B23] Porges SW Bohrer RE Cacioppo JT, Tassinary LG The analysis of periodic processes in psychophysiological research Principles of psychophysiology: physical, social, and inferential elements 1990 New York, Cambridge University Press 708 753

[B24] Heponiemi T Elovainio M Pulkki L Puttonen S Raitakari OT Keltikangas-Järvinen L Cardiac autonomic reactivity and recovery in predicting carotid atherosclerosis: the Cardiovascular Risk in Young Finns study Health Psychol 2007 26 13 21 10.1037/0278-6133.26.1.13 17209693

[B25] Raitakari OT Juonala M Rönnemaa T Keltikangas-Järvinen L Räsänen L Pietikäinen M Hutri-Kähönen N Taittonen L Jokinen E Marniemi J Jula A Telama R Kähönen M Lehtimäki T Akerblom HK Viikari JS Cohort profile: the Cardiovascular Risk in Young Finns study Int J Epidemiol 2008 37 1220 1226 10.1093/ije/dym225 18263651

[B26] Sherwood A Allen MT Fahrenberg J Kelsey RM Lovallo WR van Doornen LJ Methodological guidelines for impedance cardiography Psychophysiology 1990 27 1 23 10.1111/j.1469-8986.1990.tb02171.x 2187214

[B27] Raitakari OT Juonala M Kähönen M Taittonen L Laitinen T Mäki-Torkko N Järvisalo MJ Uhari M Jokinen E Rönnemaa T Åkerblom HK Viikari JS Cardiovascular risk factors in childhood and carotid artery intima-media thickness in adulthood: the Cardiovascular Risk in Young Finns study JAMA 2003 290 2277 2283 10.1001/jama.290.17.2277 14600186

[B28] Ravaja N Keltikangas-Järvinen L Kettunen J Cloninger's temperament dimensions and threat, stress, and performance appraisals during different challenges among young adults J Pers 2006 7 287 310 10.1111/j.1467-6494.2005.00376.x 16451233

[B29] Al'Absi M Bongard S Buchanan T Pincomb GA Licinio J Lovallo WR Cardiovascular and neuroendocrine adjustment to public speaking and mental arithmetic stressors Psychophysiology 1997 34 266 275 10.1111/j.1469-8986.1997.tb02397.x 9175441

[B30] Bongard S Mental effort during active and passive coping: a dual task analysis Psychophysiology 1995 32 242 248 7784532 10.1111/j.1469-8986.1995.tb02960.x

[B31] Porges SW Byrne EA Research methods for measurement of heart rate and respiration Biol Psychol 1992 34 93 130 10.1016/0301-0511(92)90012-J 1467397

[B32] Cheung MN Porges SW Respiratory influences on cardiac responses during attention Physiol Psychol 1977 5 53 57

[B33] Kelsey RM Guethlein W An evaluation of the ensemble averaged impedance cardiogram Psychophysiology 1990 27 24 33 10.1111/j.1469-8986.1990.tb02173.x 2339185

[B34] Brosschot JF Thayer JF Anger inhibition, cardiovascular recovery, and vagal function: a model of the link between hostility and cardiovascular disease Ann of Behav Med 1998 20 326 332 10.1007/BF02886382 10234427

[B35] Jae SY Carnethon MR Heffernan KS Choi YH Lee MK Park WH Fernhall B Slow heart rate recovery after exercise is associated with carotid atherosclerosis Atherosclerosis 2008 196 256 261 10.1016/j.atherosclerosis.2006.10.023 17137583

[B36] Lacy CR Contrada RJ Robbins ML Tannenbaum AK Moreyra AE Chelton S Kostis JB Coronary vasoconstriction induced by mental stress (simulated public speaking) Am J Cardiol 1995 75 503 505 10.1016/S0002-9149(99)80590-6 7863998

[B37] Treiber F Papavassiliou D Gutin B Malpass D Yi W Islam S Davis H Strong W Determinants of endothelium-dependent femoral artery vasodilation in youth Psychosom Med 1997 59 376 381 9251157 10.1097/00006842-199707000-00007

[B38] Sherwood A Johnson K Blumenthal JA Hinderliter AL Endothelial function and hemodynamic responses during mental stress Psychosom Med 1999 61 365 370 10367618 10.1097/00006842-199905000-00017

[B39] Dakak N Quyyumi AA Eisenhofer G Goldstein DS Cannon RO Sympathetically mediated effects of mental stress on the cardiac microcirculation of patients with coronary artery disease Am J Cardiol 1995 76 125 130 10.1016/S0002-9149(99)80043-5 7611145

[B40] Ross R The pathogenesis of atherosclerosis: a perspective for the 1990s Nature 1993 362 801 809 10.1038/362801a0 8479518

[B41] Grassi G Seravalle G Bertinieri G Turri C Stella ML Scopelliti F Mancia G Sympathetic and reflex abnormalities in heart failure secondary to ischaemic or idiopathic dilated cardiomyopathy Clin Sci 2001 101 141 146 10.1042/CS20000326 11473487

[B42] Liao D Cai J Brancati FL Folsom A Barnes RW Tyroler HA Heiss G Association of vagal tone with serum insulin, glucose, and diabetes mellitus - the ARIC Study Diabetes Res Clin Pract 1995 30 211 221 10.1016/0168-8227(95)01190-0 8861461

[B43] Balleshofer BM Rittig K Enderle MD Volk A Maerker E Jacob S Matthaei S Rett K Haring HU Endothelial dysfunction is detectable in young normotensive first-degree relatives of subjects with type 2 diabetes in association with insulin resistance Circulation 2000 101 1780 1784 10769277 10.1161/01.cir.101.15.1780

[B44] Li J Zhao SP Li XP Zhuo QC Gao M Lu SK Non-invasive detection of endothelial dysfunction in patients with essential hypertension Int J Cardiol 1997 61 165 169 10.1016/S0167-5273(97)00153-8 9314210

[B45] Rozanski A Kubzansky LD Psychologic functioning and physical health: a paradigm of flexibility Psychosom Med 2005 67 Suppl 1 S47 53 10.1097/01.psy.0000164253.69550.49 15953801

[B46] Lucini D Norbiato G Clerici M Pagani M Hemodynamic and autonomic adjustments to real life stress conditions in humans Hypertension 2002 39 184 188 10.1161/hy0102.100784 11799100

[B47] Huang PH Leu HB Chen JW Cheng CM Huang CY Tuan TC Ding PY Lin SJ Usefulness of attenuated heart rate recovery immediately after exercise to predict endothelial dysfunction in patients with suspected coronary artery disease Am J Cardiol 2004 93 10 13 10.1016/j.amjcard.2003.09.004 14697458

[B48] Houtveen JH Rietveld S De Geus EJ Contribution of tonic vagal modulation of heart rate, central respiratory drive, respiratory depth, and respiratory frequency to respiratory sinus arrhythmia during mental stress and physical exercise Psychophysiology 2002 39 427 436 10.1017/S0048577202394022 12212635

[B49] Berntson GG Bigger JT Jr Eckberg DL Grossman P Kaufmann PG Malik M Nagaraja HN Porges SW Saul JP Stone PH Molen MW van der Heart rate variability: origins, methods, and interpretive caveats Psychophysiology 1997 34 623 648 10.1111/j.1469-8986.1997.tb02140.x 9401419

